# Plasmodium Vivax in a Healthy, Young Immigrant Male

**DOI:** 10.7759/cureus.65743

**Published:** 2024-07-30

**Authors:** Ami Patel, Diahann Marshall

**Affiliations:** 1 Family Medicine, Louisiana State University Health Shreveport, Alexandria, USA; 2 Family Medicine, Rapides Regional Medical Center, Alexandria, USA

**Keywords:** vivax parasite, hispanic immigrants, international and travel medicine, plasmodium vivax malaria, malaria infection

## Abstract

A young immigrant male presented to the hospital with multiple complaints and was found to be in septic shock with symptomatic anemia. After an extensive workup, the patient was found to have malaria, a disease caused by the bite of an infected mosquito. This case outlines the pertinent findings, relevant diagnostic tests, and appropriate treatment for a patient with malaria secondary to Plasmodium vivax. It also demonstrates the importance of social and travel history, especially when evaluating our immigrant populations.

## Introduction

Occurring mostly in tropical climates and originating from the genus Plasmodium, malaria is a life-threatening parasitic disease transmitted from infected Anopheles mosquitoes to humans [1]. Although malaria is not frequently encountered in the United States, it is essential to identify and treat it promptly. It is a preventable and curable disease, with symptoms ranging from mild to severe [1]. One of the setbacks in management is that the medications for treatment are seldom available within most hospital settings in the United States. This case report describes a presentation of malaria in a young adult male who traveled from South America.

## Case presentation

A healthy 25-year-old male with no past medical history presented to an outside hospital with complaints of weakness, poor appetite, nausea, and melena. He was then transferred for a higher level of care after he was found to have symptomatic bradycardia, hypotension, anemia, and a concern for GI bleed. Further history revealed that the patient had traveled from Honduras to Ecuador by land and then to the United States by boat over a few weeks. He stated that he was doing fine until the boat ride, after which he began to have weakness, melena, and poor appetite. He reported that while on the boat, the only medication he took was dimenhydrinate for motion sickness. He was traveling with unknown individuals whose medical histories were unavailable. The patient was placed under Immigration and Customs Enforcement (ICE) care upon his arrival, and his symptoms persisted. He was monitored at the ICE facility for five days and found to be bradycardic with a heart rate in the 40 beats per minute range and hypotensive with a blood pressure around 70/30 mmHg.

He was taken to the nearest Emergency Department, where his hemoglobin levels were less than seven g/dL (normal 13.6 to 17.7 g/dL). The patient was given two units of packed red blood cells (PRBCs) before transfer. On arrival, his initial vital signs were oral temperature of 98.0 degrees Fahrenheit (36.7 degrees Celsius), pulse rate of 44 beats per minute, respiratory rate of 15 breaths per minute, blood pressure of 101/71 mmHg, and oxygen saturation of 99% on room air by pulse oximetry. Initial laboratory findings showed that he had normocytic anemia, thrombocytopenia, mild hyperglycemia, elevated total bilirubin with normal aspartate aminotransferase (AST) and alanine transaminase (ALT), and otherwise unremarkable results (Table 1). During this time, the patient remained alert, awake, and oriented, with no acute changes in his mental status. He was initially admitted for septic shock, bradycardia, hypotension, and symptomatic anemia.

**Table 1:** Pertinent initial laboratory findings

	Results	Reference range
White Blood Cell Count	5.2	5.0 - 10.0 K/mm^3^
Hemoglobin	10.2	12.0 - 16.0 gm/dl
Hematocrit	31.3	40.0 - 54.0%
Mean Corpuscular Volume	85.8	80.0 - 100.0 fl
Platelet Count	99	150 - 450 K/mm^3^
Sodium	134	135 - 148 mmol/l
Potassium	4.1	3.3 - 5.1 mmol/l
Chloride	107	98 - 107 mmol/l
Corrected Calcium	8.9	8.4 - 10.7 mg/dl
Blood Urea Nitrogen	7	6 - 19 mg/dl
Creatinine	0.75	0.70 - 1.30 mg/dl
Glucose	136	70 - 120 mg/dl
Total Bilirubin	1.6	0.0 - 1.0 mg/dl
Aspartate Transferase	25	0 - 37 units/l
Alanine Transaminase	14	0 - 40 units/l
Total Alkaline Phosphate	99	40 - 130 units/l
Lactic Acid	0.9	0.7 - 2.5 mmol/l

**Figure 1:**
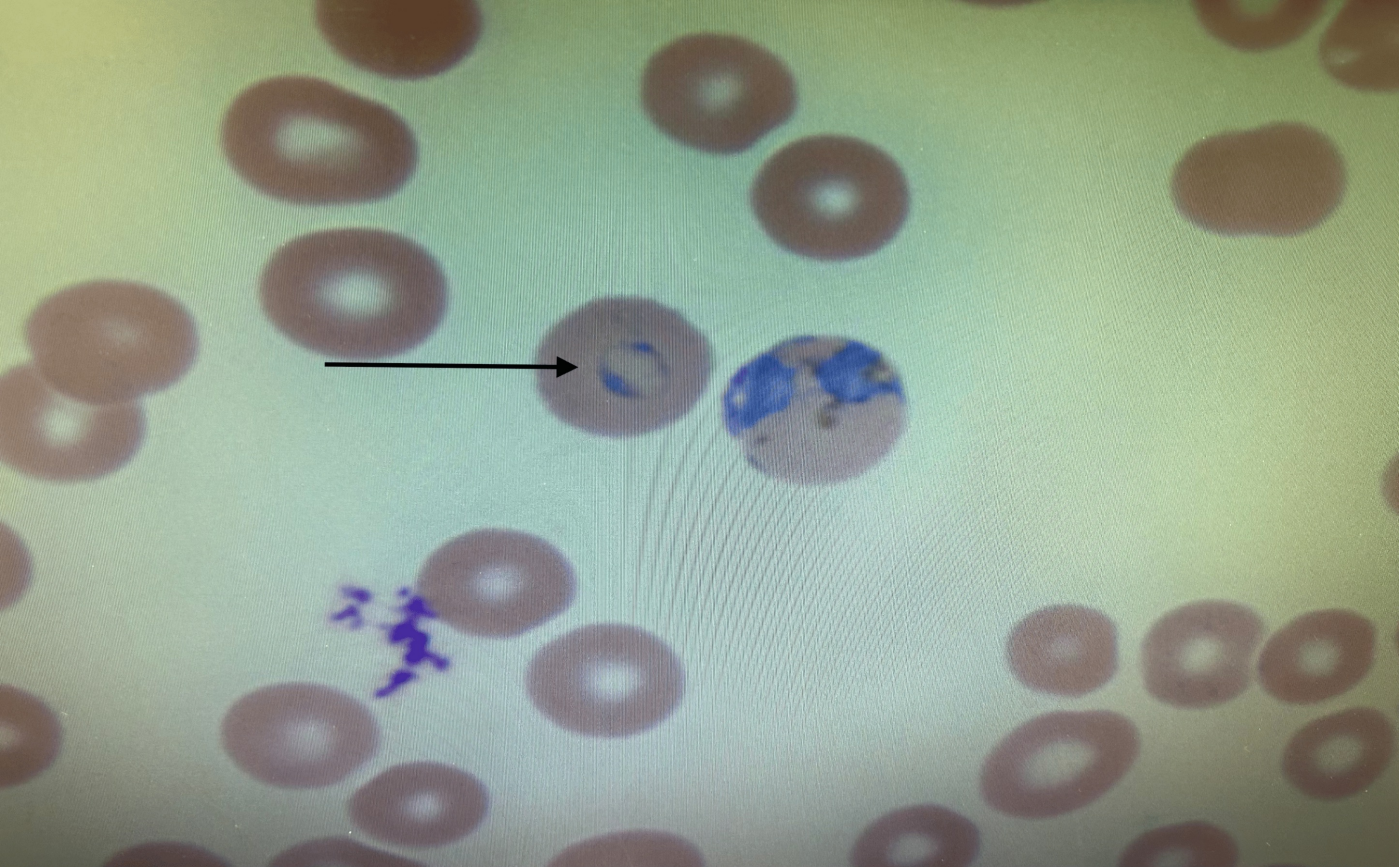
Peripheral blood smear showing ring trophozoite formation

Cardiology and Gastroenterology specialists were consulted to assist with the patient's care. The cardiologist's workup was unremarkable, with the conclusion that his bradycardia and hypotension were likely secondary to sepsis. His fecal occult blood test was negative from his gastroenterology workup, and imaging demonstrated splenomegaly. His hemoglobin remained stable, and no additional episodes of blood loss were reported, so the gastroenterologist decided to manage conservatively while the rest of his workup was ongoing. The patient developed a fever on hospital day two, then again 24 hours later. His highest temperature was recorded on hospital day three at 101.3 degrees Fahrenheit (38.5 degrees Celsius). Meeting septic shock criteria, he was started on broad-spectrum intravenous antibiotics with vancomycin and cefepime pending further infectious workup. Urinalysis, chest radiography, blood, and stool cultures were unremarkable for infectious sources. Considering where he recently traveled, malaria thick and thin smears were ordered and were positive for Plasmodium species with 1.0-1.9% parasite density/count (Figure 1).

An infectious disease specialist was consulted, and it was recommended that treatment be initiated with artemether-lumefantrine, atovaquone-proguanil, or quinine plus doxycycline. Artemether-lumefantrine and atovaquone-proguanil were not readily available in the hospital pharmacy and needed to be ordered from an outside source. The patient was immediately started on a quinine plus doxycycline regimen due to the anticipated delay in obtaining one of the other two regimens. He received quinine and doxycycline for 24 hours but was transitioned to atovaquone-proguanil when it became available. The patient completed the recommended three-day course of atovaquone-proguanil, and his blood sample was sent for Plasmodium polymerase chain reaction (PCR) testing to help determine the subtype of species. The malaria thick and thin smears were repeated every 12 hours with a parasite density count until a negative test resulted. Glucose-6-phosphate-dehydrogenase (G6PD) testing was also done considering the side effects of anti-malarial drugs. During this time, the patient was closely monitored for mental status changes, hypoglycemia, acute renal failure, worsening anemia, and metabolic acidosis. The Plasmodium PCR returned as Plasmodium vivax, and G6PD testing was within normal limits. Throughout his hospitalization, the patient's symptoms, bradycardia, and hypotension improved. He was discharged back to the ICE facility with the appropriate and recommended treatment for Plasmodium vivax, which was primaquine 30 mg base (52.6 mg total) daily for 14 days.

## Discussion

Malaria is transmitted via infected mosquito bites and is caused by Plasmodium parasites. It is the leading cause of illness and death in many tropical and subtropical regions, primarily in Africa, South Asia, and parts of Central and South America. It is a preventable and curable disease, but if left untreated, it can lead to severe anemia, neurological damage, and even death [2]. According to the World Health Organization, 249 million cases of malaria were reported worldwide, with 608,000 deaths in the year 2022 [1]. According to the Centers for Disease Control and Prevention (CDC), around 2,000 cases are diagnosed in the United States yearly, with most of the cases occurring in people who contract the disease while traveling in an endemic region [2]. Of the five Plasmodium species, Plasmodium vivax is the most widespread, predominantly found in South America and Southeast Asia. The Anopheles mosquito vector spreads the parasite. Able to survive in various climates due to its dormant state, its geographical range is more widespread than other malaria species [3].

Its transmission occurs when formation sporozoites from the mosquito are transmitted into a human host and immediately invade the hepatocytes, forming schizonts. The rupture of the schizonts releases Plasmodium merozoites into the blood; the stage that coincides with symptoms of malaria. Of the five malaria species, only Plasmodium vivax and ovale have hypnozoites that can remain dormant in the liver for months to years. This prolonged contamination can lead to a relapse of infection when the hypnozoites re-enter the blood [3-4]. Interestingly, Plasmodium vivax merozoites only infect reticulocytes, unlike its other Plasmodium counterparts, which infect all stages of a red blood cell. Although this results in lower parasitemia levels, typically not exceeding 2-3%, the exclusivity of infecting reticulocytes can lead Plasmodium vivax to result in significant disease because of its increased host immune response [5].

The diagnosis should include clinical assessment, laboratory testing, and a thorough social history to identify recent travel. If a patient is from or recently traveled to an endemic area, suspicion of malaria should be raised as a possible differential. Some other differential diagnoses are influenza, dengue fever, chikungunya, typhoid fever, bacteremia, leptospirosis, rickettsia, and viral hepatitis. Symptoms can include fever, chills, flu-like illness, headache, muscle aches, fatigue, joint pain, sweating, nausea, and vomiting [1-2]. Laboratory testing typically involves blood smears to identify parasites and is the gold standard [6]. Treatment is based on multiple factors, including the patient's clinical status, the species type, the area where the infection was acquired, the drug-resistance status, and the use of previous antimalarial agents [7]. Overall, the prognosis of a Plasmodium vivax infection is favorable if identified and treated appropriately. However, complications can occur, leading to more severe diseases such as cerebral malaria, renal failure, acute respiratory failure, and shock [3].

In this case, the patient was found to be infected with Plasmodium vivax, which requires additional treatment for the hypnozoites, which can cause relapse due to its dormant state in the liver. Plasmodium vivax has been reported to have different drug resistance patterns based on the geographic region from where it has been acquired. Typically, the effective treatment of choice is chloroquine [2-3]. This patient came from South America, where rare cases of chloroquine-resistant Plasmodium vivax have been documented. With this in mind, treatment options included artemether-lumefantrine, atovaquone-proguanil, or quinine plus doxycycline or tetracycline for the acute phase of malaria [2-3].

Quinine plus doxycycline was initially used for this patient as the other two options were not readily available. As soon as atovaquone-proguanil was obtained, the patient was started on a three-day course of the medication. The change in regimen was made due to quinine's side effect profile, which includes arrhythmias.

Treatment for the hypnozoites is with either primaquine phosphate or tafenoquine. Both of these can cause hemolytic anemia in G6PD deficiency, so quantitative testing should be done prior to initiation of treatment [3]. This patient had normal G6PD levels, so he was discharged on primaquine for 14 days.

Travel education is essential and integral to conversations with patients who plan on visiting endemic regions. Many factors can help reduce the risk of malaria transmission. These range from appropriate medication prophylaxis to wearing loose-fitting, long-sleeved shirts and pants to the use of diethyltoluamide (DEET) or permethrin and to behavioral modifications such as knowing that mosquitoes generally bite around dawn and dusk [3]. Adhering to some of these precautions can help reduce the risk of acquiring and spreading the infection.

## Conclusions

Identifying and diagnosing malaria in patients presenting with symptoms and a travel history to and from endemic regions is challenging but important. Early initiation of treatment is vital, as it can prevent serious complications, including death, from the disease. This case highlights the importance of obtaining a good history of presenting illness, inquiring about travel history when appropriate, broadening the differential diagnoses, and considering possible causes of a patient's presentation outside of the more common diseases found in the United States.
